# Spinal Irritation

**Published:** 1837

**Authors:** 

**Affiliations:** Cumminsville, Ohio; Cumminsville, Ohio


### Art. VI.—Spinal Irritation.

*Cases of Spinal Irritation successfully treated.* By Drs. Mount and Bamford of Cumminsville, Ohio; reported by John Bamford, M. D.

Case 1. A lady aged forty-three, of nervous temperament, the mother of six children, had been afflicted for seven years with various anomalous complaints, simulating organic affections of the thoracic and abdominal viscera; and had been treated by a number of professional gentlemen without much relief. About the first of March, 1836, she applied for medical advice. At this time she labored under pain in different parts of the chest, palpitation of the heart, difficult and laborious respiration, increased on the least exertion; dry and harrassing cough, pain in the head with throbbing of the temporal arteries, pain and soreness of the stomach with acidity, pyrosis, etc. Appetite bad, tongue furred, bad taste in the mouth, bowels torpid, abdomen painful and tender on pressure, functional derangement, of the liver, dysmenorrhea, Leucorrhœa and a sad train of symptoms generally denominated nervous and hysterical, with general debility; tenderness from pressure along the whole course of the spinal column; pressure made on the inferior portion of the cervical and superior dorsal portion increased the pain in the breast and stomach, and produced violent paroxysms of coughing.

The treatment was directed pricipally to the spine, and consisted in the frequent application of cups with scarification, followed by blisters, tartar emetic ointment, etc. Internal remedies were administered to meet other indications. This course was pursued for the space of nearly four months, when the spinal tenderness was removed completely, and with it all the other morbid symptoms.

Case 2. A young lady about twenty-three years of age, had been afflicted for a long time with pain in the thorax, cough, difficult respiration, palpitation of the heart, headache, various nervous and dyspeptic symptoms, loss of appetite, langor and debility, for which various medicines had been tried without permanent relief. On examination, found tenderness of the cervical and dorsal portion of the spine, and discovered that pressure produced acute pain in the head and chest, with violent fits of coughing.

From the frequent application of cups with scarification, and counter irritation to the affected portion of the spine, together with medicines to correct the disordered state of the stomach and bowels, the distressing symptoms all vanished.

Case 3. A lady aged thirty-five, of delicate constitution, the mother of seven children, had been afflicted for nine years, with pain in the breast along the sternum, and in the left side. Her own account of her severe and protracted sufferings, was, that three weeks after confinement with her second child, she had an attack of fever, and ever since has been troubled more or less with pain in the breast. About three years ago, after the birth of another child, she was taken with a severe vomiting, which increased the pain in the breast, and produced prolapsus uteri; since that time she has been afflicted with dysmenorrhea, leucorrhœa, functional derangement of the stomach and liver, pain in the breast, palpitation of the heart, difficult respiration, cough, etc., with numbness of the right arm. She had been treated by a number of medical men and taken a great deal of medicine without relief, was blistered frequently on the breast, stomach and sides, and subjected to different counter irritants, over the seat of pain without any benefit.

She says that no two physicians agreed as to the nature of her disease, some supposing her to have consumption, others, a disease of the liver, others a disease of the spleen, others of the heart, etc., and each one treating her, of course, according to his views of the pathology of the disease. In the summer of 1835, she had an attack of bilious fever, and was severely salivated by her medical attendant; her gums and cheeks sloughed, and adhesions took place, so that she cannot open her mouth more than to the extent of three-fourths of an inch, and is unable to protrude her tongue beyond her lips.

She became pregnant, and on the ninth of March, 1836, in the fourth month of her pregnancy aborted, and suffered from an attack of peritonitis, with an aggravation of all her former symptoms; blood was drawn from the arm, purgatives were administered, and warm fomentations applied to the abdomen, with partial relief to the peritoneal inflammation; stomach became very irritable, rejecting every thing that was taken, the other symptoms more aggravated. On examination the spine was found tender along its whole course; applied cups with scarification which relieved the urgent symptoms, in the thoracic region. Blisters were tried, with relief of the abdominal symptoms; in a few days pain in the head was experienced, with a return of the pain in the breast, and palpitation of the heart; pressure on the superior cervical vertebræ increased the pain in the head, and on the inferior cervical and superior dorsal vertebræ, increased that in the breast, and excited severe coughing; drew blood from the cervical and dorsal portion of the spine, with cups to the amount of xiiℨ which relieved the symptoms; the bowels were opened with calcined magnesia, the only laxative that could be retained on the stomach; tried antispasmodics and anodynes to quiet nervous irritability, but they were all rejected.

On the first of April, she was again attacked with severe pain in the head and breast, with difficult and hurried respiration, was bled from the arm to the amount of xℨ which relieved the head and respiration, ordered calcined magnesia to move the bowels. The symptoms still continued, though somewhat moderated. On the 10th drew xvℨ of blood by means of cups from the spine, when she became faint and continued quite exhausted all day, and had distressing naseua; ordered her small quantities of brandy at short intervals, alternated with small portions of cinchona, columbo, cinnamon and mineral acids; by persisting in the use of these medicines, the system rallied, the stomach was relieved, and with a light vegetable diet her health improved.

The abstraction of blood was now desisted from, and tartar emetic and blisters were repeatedly applied, along the whole course of the spine with decided advantage; vegetable tonics with carb. fer. were administered, tinct. canth. and astringent injections were employed, for the leucorrhœa.

This treatment was strictly adhered to, until the first of May, when she was able to sit up, and in a few days more to go about the house, and attend to the duties of her family. On the 12th was still going about, but complained of pain in the breast and numbness of the right arm, tenderness of the spine under pressure on the inferior cervical and superior dorsal portion. Applied cups with scarification, to the affected part, which relieved both affections.

Case 4. A lady twenty-seven years of age, of bilious temperament, the mother of two children, now a widow; for nine years had been troubled with a variety of nervous disorders, with functional derangements of the stomach and liver, dysmenorrhœa, leucorrhœa, etc.; and at every menstrual period had epilepsy,—tenderness of spine on pressure.

On the ninth of March, 1836, she had an attack of icterus, when she applied for medical advice. The jaundice was treated in the usual method, but did not yield to remedies until they were applied to the spine.

Blood was drawn from the spine by means of cups with scarification, and pain relieved. Ordered a purging draught. The next day, the symptoms were more favorable, bowels becoming regular, skin loosing its yellow tinge, appetite returning, ordered a light vegetable diet, laxatives, rubefacients and frictions of the spine, and to the hepatic and gastric regions.

On the 30th, from exposure to a cold and damp atmosphere and imprudence in diet, became affected with profuse diarrhœa, dejections light colored and offensive, abdomen swollen and tender on pressure, complained of pain in the pelvic region, and sickness of stomach, spine tender. Applied cups with scarification to the lumbar and dorsal portions of the spine; ordered small doses of calomel and opium with ipecacuanha, allowed no nourishment but mucilage of gum arabic. On the 4th of April, diarrhœa checked, ordered a dose of calomel to be followed by castor oil; and, as she thought herself threatened with an epileptic attack, ordered a blister to the spine and gave antispasmodics. On the 6th, bowels had been freely opened, pain, soreness, and swelling gone. Complains of nothing but the blistered surface, appetite improving; ordered a light vegetable diet, tinc. mur. feri. and laxatives. 10th. Still improving, complains of slight pain in the back, with distress in the pelvic region; ordered a brisk cathartic, pediluvium, etc. Continue tinc. mur. ferri. 12th, Catamenia took place, attended with pain, but without epilepsy. 14th. Health improving, can detect slight tenderness in some portions of the spine; recommended the occasional application of tartar emetic or a blister to the spine, and astringent injections for the leucorrhœa, continue tinc. ferri.; she left the neighborhood, the next day, in fine spirits. We have not heard from her since.

Case 5. A lady aged thirty-two, of robust constitution, the mother of four children, had been in ill health for some time, and in the fall of 1835, had intermittent fever, which left her health quite impaired.

Was called to visit her on the 10th of March, 1836. She complained of pain in the head, and pain in the stomach with tenderness on pressure; bowels constipated, tongue coated towards the root, with a thick white fur, red at tip; pain in the left side, difficult respiration, frequent sighing, appetite bad, general lassitude, pain in the back, cold extremities, dysmenorrhœa, leucorrhœa, etc., the pulse full and corded.

Prescribed venesection and mild purgatives; the symptoms appeared more favorable for a few days. On the 14th, had a a chill, followed by fever, which aggravated all the former symptoms, and terminated in regular paroxysms of intermittent fever, of the tertian type; on examination, found the spine tender throughout its whole course; applied cups with scarification to the spine, followed by a blister; gave alterative doses of mercury with mild laxatives, and sulphate of quinin which soon relieved the intermittent; and, as the tenderness of the spine gave way to topical applications, the other symptoms subsided.

About the first of May she had another paroxysm of intermittent, with pain in the epigastric and left hypochondriac regions, increased from pressure on the back, applied cups to the spine, followed by a tarter emetic plaster; gave mild mercurials and quinin. 6th. Free from pain and soreness, general health improving, gave tinc. canth. and ordered astringent injections, per vaginam, for the leucorrhœa. 14th, Pain and soreness of breast and stomach, tenderness of the superior dorsal portion of the spine; applied cups which relieved the stomach and breast; continued other medicines as before, applied tartar ointment to the whole course of the spine. About the first of June no pain, appetite good, bowels regular, leucorrhœa nearly well, says she feels better than she has for a year past. The intermittent in this case was evidently kept up by the spinal disease.

Case 6. A young lady aged 26, applied for advice about the 10th of March, 1836. She stated that she had been in ill health for three years. The symptoms at the time of application, were pain in the head, with soreness of the integuments, particularly of the occipital portion, pain and soreness of the epigastric and hepatic region, pain under the left mammary gland, tongue covered with a dirty heavy fur, disagreeable taste in the mouth, sallow complexion, countenance expressive of severe suffering, lassitude, coldness of the extremities, loss of appetite, dysmenorrhea, leucorrhœa, etc.

Tenderness of spine on pressure. Applied cups with scarification to the dorsal portion, and ordered a mercurial cathartic and a pediluvium of the nitro-muriatic acid mixture, with friction to the epigastric and right hypochondriac regions with the same mixture; visited her on the 16th, the medicine had operated freely, and carried off a large quantity of vitiated secretion from the bowels, tongue still foul, countenance more cheerful, pain and soreness of the epigastric and hepatic regions much relieved, pain in the head and left side continuing as before. Drew xv℥ of blood from the cervical and superior dorsal portion of the spine, gave alterants and mild laxatives, ordered a blister to be applied to the whole course of the spine in a day or two. 26th. Complains of no pain or soreness, except slight aching in the head. Ordered a blister to the back of the neck, prescribed light tonics, continue alterants and laxative. May 5th, health improving, some tenderness of spine; applied tartar emetic to the affected portion, and ordered muriated tincture of iron. 20th, Health still improving, all the morbid symptoms have vanished, declined taking any more medicine.

*February,* 1837.

